# P-1682. Accuracy of Xpert MTB/XDR Panel to Detect Antitubercular Drug Resistance in Indian Children

**DOI:** 10.1093/ofid/ofaf695.1856

**Published:** 2026-01-11

**Authors:** Dhruv Gandhi, Shatakshi Garg, Sonal Patil, Dhruv Mamtora, Ira Shah

**Affiliations:** Bai Jerbai Wadia Hospital for Children, Mumbai, India, West Monroe, LA; Bai Jerbai Wadia Hospital for Children, Mumbai, India, West Monroe, LA; Bai Jerbai Wadia Hospital for Children, Mumbai, India, West Monroe, LA; Bai Jerbai Wadia Hospital for Children, Mumbai, India, West Monroe, LA; Bai Jerbai Wadia Hospital for Children, Mumbai, India, West Monroe, LA

## Abstract

**Background:**

The microbiological diagnosis of tuberculosis (TB) has evolved from conventional methods like culture and phenotypic drug-sensitivity testing (pDST) to rapid molecular assays such as line probe assays (LPA), Xpert MTB/Rif, Xpert Ultra, and Xpert MTB/XDR. While LPAs are highly sensitive and specific, they require specialized infrastructure and training, limiting their use in resource-constrained settings. Xpert MTB/XDR, a newer reflex test, detects resistance to multiple drugs, but its performance in pediatric TB remains understudied. The aim of this study is to evaluate the agreement between Xpert MTB/Rif or Ultra, Xpert MTB/XDR, LPAs, and 3-drug pDST for detecting antitubercular drug resistance in children.

**Table 1:**
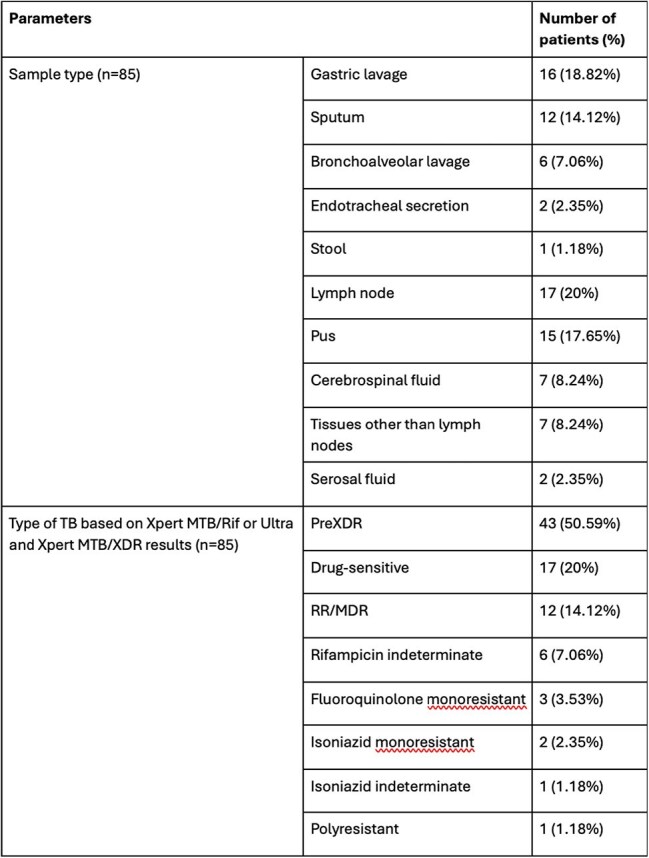
Type of sample tested and the type of TB diagnosed in the patients Note: TB- Tuberculosis, XDR- Extensively drug-resistant, RR- Rifampicin resistant, MDR- Multidrug-resistant.

**Table 2:**
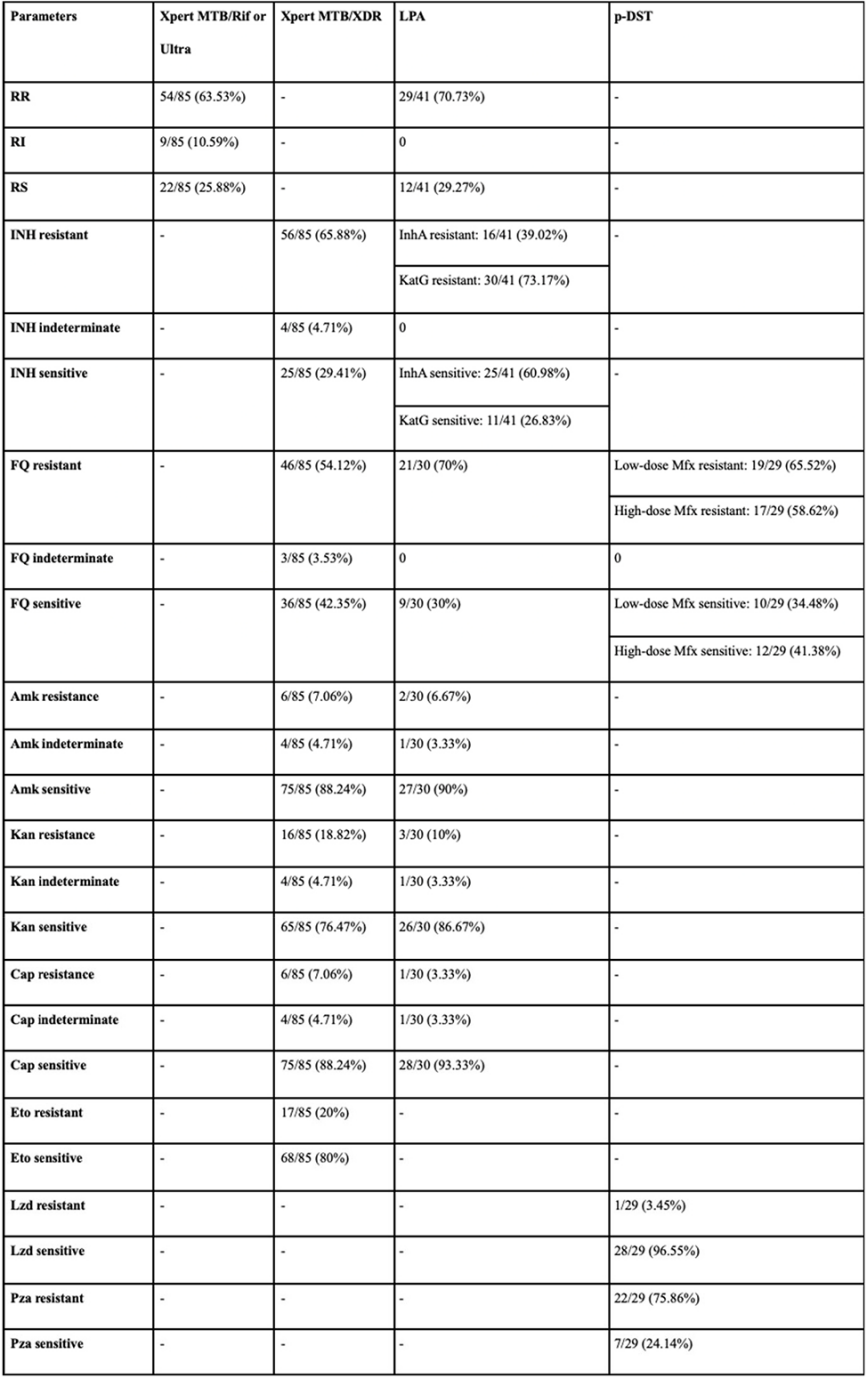
Results of antitubercular drug resistance on Xpert MTB/Rif or Ultra, Xpert MTB/XDR, first- and second-line LPA, and 3-drug pDST. Note: LPA- Line probe assay, pDST- Phenotypic drug sensitivity test, RR- Rifampicin resistant, RI- Rifampicin indeterminate, RS- Rifampicin sensitive, INH- Isoniazid, FQ- Fluoroquinolone, Mfx- Moxifloxacin, Amk- Amikacin, Kan- Kanamycin, Cap- Capreomycin, Eto- Ethionamide, Lzd- Linezolid, Pza- Pyrazinamide.

**Methods:**

A retrospective study was conducted at a tertiary children's hospital in Mumbai, India. We including 85 children less than 18 years of age, diagnosed with TB, between October 2020 and September 2024. Pulmonary and extrapulmonary samples were tested using Xpert MTB/Rif or Ultra, Xpert MTB/XDR, LPAs (first- and second-line), and 3-drug pDST (pyrazinamide, linezolid, moxifloxacin). Kappa agreement was determined between these investigations for the respective drug-resistance results.

**Table 3:**
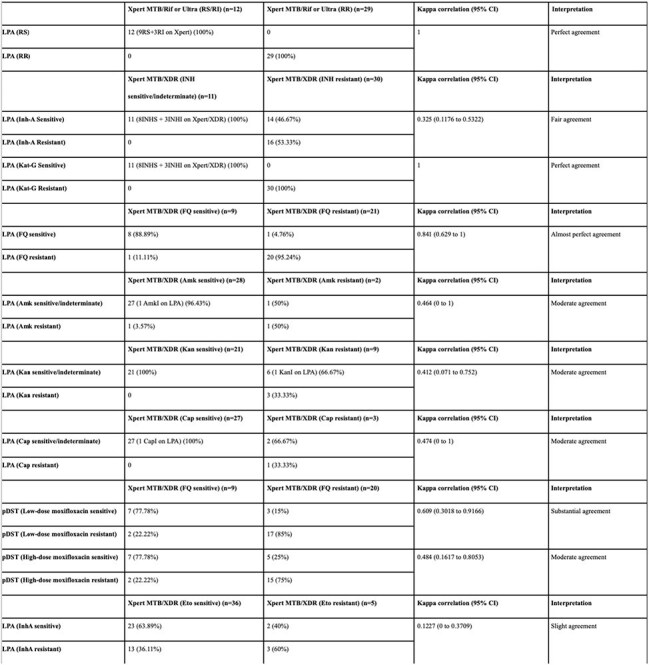
Kappa correlation between Xpert MTB/Rif or Ultra and first-line LPA for rifampicin resistance results, between Xpert MTB/XDR and first- and second-line LPA for the corresponding drug resistance results, between Xpert MTB/XDR and pDST for fluoroquinolone resistance results, and between Xpert MTB/XDR results for ethionamide resistance and InhA resistance results on LPA. Note: RS- Rifampicin sensitive, RI- Rifampicin indeterminate, RR- Rifampicin resistant, CI- Confidence interval, LPA- Line probe assay, pDST- Phenotypic drug susceptibility test, INH- Isoniazid, INHS- Isoniazid sensitive, INHI- Isoniazid indeterminate, FQ- Fluoroquinolone, Amk- Amikacin, AmkI- Amikacin indeterminate, Kan- Kanamycin, KanI- Kanamycin indeterminate, Cap- Capreomycin, CapI- Capreomycin indeterminate, Eto- Ethionamide.

**Results:**

Perfect agreement was observed between Xpert MTB/Rif or Ultra and LPA for rifampicin resistance(κ=1). Xpert MTB/XDR showed perfect concordance with LPA for *katG*-mediated isoniazid resistance(κ=1) but only fair agreement for *inhA*-mediated isoniazid resistance(κ=0.325). Xpert MTB/XDR showed an almost perfect agreement with LPA for fluoroquinolone resistance(κ=0.841), with substantial(κ=0.609) and moderate(κ=0.484) agreement for low- and high-level moxifloxacin resistance on pDST, respectively. Moderate agreement was noted for second-line injectable drugs (amikacin, kanamycin, capreomycin) between Xpert MTB/XDR and LPA. Slight agreement(κ=0.1227) was found between ethionamide resistance on Xpert MTB/XDR and *inhA* resistance on LPA.

**Conclusion:**

Xpert MTB/XDR shows a good degree of concordance for drug resistance results with first- and second-line LPA and moxifloxacin pDST in Indian children except for ethionamide. It may be considered as a standalone test to determine the type of drug-resistant TB and devise a second-line regimen upfront.

**Disclosures:**

All Authors: No reported disclosures

